# The suitability of zebrafish (danio rerio) as an optimal organism to further investigate the associated schizophrenia-like phenotype

**DOI:** 10.1192/j.eurpsy.2023.945

**Published:** 2023-07-19

**Authors:** I. B. Nita, O. D. Ilie, A. S. Ciobica, L. D. Hritcu, C. Solcan, R. P. Dobrin

**Affiliations:** 1 Department of Medicine III, “Grigore T. Popa” University of Medicine and Pharmacy; 2 Department of Biology, “Alexandru Ioan Cuza” University; 3 Internal Medicine Clinic; 4 Department of Molecular Biology, Histology and Embryology, “Ion Ionescu de la Brad” University of Life Sciences, Iasi, Romania

## Abstract

**Introduction:**

Zebrafish (*Danio rerio*) has evolved over the years as a preferred organism due to its vast repertoire in research fields that mimic a targeted phenotype, particularly behavioral typologies and specific attributes comparable to murine models and relatively high homology with humans. Considering this consideration, different pharmacological treatments have been tested and proven that under different concentrations may trigger schizophrenia (SCZ)-like symptoms.

**Objectives:**

Starting from the actual stage of knowledge according to which agents used as N-methyl-D-aspartate (NMDA) inhibitors (MK-801, ketamine, and phencyclidine) alongside psychedelic (mescaline, lysergic acid diethylamide), psychoactive substances (amphetamine), and non- and essential amino acids (proline and methionine), we aimed to reunite and review all existing evidence. This approach may offer an updated and critical overview regarding the possible future directions surrounding these compounds regarding the pharmaco-dynamics/kinetics.

**Methods:**

To ensure the coverage of all relevant literature, we performed searches in four databases (PubMed/Medline, ISI Web of Knowledge, Scopus, and ScienceDirect) per combinations of keywords: “schizophrenia” with “MK-801”, “ketamine”, “phencyclidine”, “mescaline”, “lysergic acid diethylamide”, “amphetamine”, “proline”, “methionine”, and “zebrafish”. Eligible studies had to be “research article(s)” written in English.

**Results:**

A total of n = 246 studies were returned during the established interval (2010 – 2022). Precisely, n = 16 were identified in PubMed/Medline, n = 16 in ISI Web of Knowledge, n = 17 in Scopus, and n = 197 in ScienceDirect per database. If taken per year, n = 13 were published in 2010, n = 12 in 2011, n = 15 in 2012, n = 24 in 2013, n = 21 in 2014, n = 21 in 2015, n = 32 in 2016, n = 8 in 2017, n = 12 in 2018, n = 9 in 2019, n = 17 in 2020, n = 34 in 2021, and n = 28 in 2022. Depending on the combination of keywords, we had the following situation: n = 65 for “schizophrenia+MK-801+zebrafish”, n = 42 for “schizophrenia+ketamine+zebrafish”, n = 21 for “schizophrenia+phencyclidine+zebrafish”, n = 4 for “schizophrenia+mescaline+zebrafish”, n = 5 for “schizophrenia+lysergic acid diethylamide+zebrafish”, n = 36 for “schizophrenia+amphetamine+zebrafish”, n = 37 for “schizophrenia+proline+zebrafish”, n = 36 for “schizophrenia+ methionine+zebrafish”.

**Conclusions:**

There can be seen an uprising trend in the current literature of studies focused on the administration of MK-801, ketamine, amphetamine, proline, methionine, and phencyclidine aiming to trigger SCZ-like symptoms as opposed to mescaline and lysergic acid diethylamide. Most of the data is contradicting, with either a decrease/increase in behavior (locomotion, aggression, sociability, circling behavior, and memory deficits), which is why additional studies are mandatory.

**Disclosure of Interest:**

None Declared

